# Tailoring Care to Vulnerable Populations by Incorporating Social Determinants of Health: the Veterans Health Administration’s “Homeless Patient Aligned Care Team” Program

**DOI:** 10.5888/pcd13.150567

**Published:** 2016-03-31

**Authors:** Thomas P. O’Toole, Erin E. Johnson, Riccardo Aiello, Vincent Kane, Lisa Pape

**Affiliations:** Author Affiliations: Erin E. Johnson, Riccardo Aiello, Lisa Pape, The National Center on Homelessness Among Veterans, Office of Homeless Programs, US Department of Veterans Affairs, Providence, Rhode Island; Vincent Kane, The National Center on Homelessness Among Veterans, Office of Homeless Programs, US Department of Veterans Affairs, Providence, Rhode Island and Lebanon VA Medical Center, Lebanon, Pennsylvania.

## Abstract

**Introduction:**

Although the clinical consequences of homelessness are well described, less is known about the role for health care systems in improving clinical and social outcomes for the homeless. We described the national implementation of a “homeless medical home” initiative in the Veterans Health Administration (VHA) and correlated patient health outcomes with characteristics of high-performing sites.

**Methods:**

We conducted an observational study of 33 VHA facilities with homeless medical homes and patient- aligned care teams that served more than 14,000 patients. We correlated site-specific health care performance data for the 3,543 homeless veterans enrolled in the program from October 2013 through March 2014, including those receiving ambulatory or acute health care services during the 6 months prior to enrollment in our study and 6 months post-enrollment with corresponding survey data on the Homeless Patient Aligned Care Team (H-PACT) program implementation. We defined high performance as high rates of ambulatory care and reduced use of acute care services.

**Results:**

More than 96% of VHA patients enrolled in these programs were concurrently receiving VHA homeless services. Of the 33 sites studied, 82% provided hygiene care (on-site showers, hygiene kits, and laundry), 76% provided transportation, and 55% had an on-site clothes pantry; 42% had a food pantry and provided on-site meals or other food assistance. Six-month patterns of acute-care use pre-enrollment and post-enrollment for 3,543 consecutively enrolled patients showed a 19.0% reduction in emergency department use and a 34.7% reduction in hospitalizations. Three features were significantly associated with high performance: 1) higher staffing ratios than other sites, 1) integration of social supports and social services into clinical care, and 3) outreach to and integration with community agencies.

**Conclusion:**

Integrating social determinants of health into clinical care can be effective for high-risk homeless veterans.

## Introduction

In 1988, the Institute of Medicine described homeless-related health problems as 3-pronged: health problems caused by homelessness, health problems that cause homelessness, and health conditions that are difficult to treat because of homelessness (1). Research has documented the significant disease burden among the homeless population (2,3); one community-based survey found that 66% of the homeless had a chronic medical problem, and 33% had 2 or more mental health problems (4). Homeless people have a 3.5 times higher age-adjusted mortality than their domiciled counterparts (5); the average age of death among homeless people is the mid-50s (6,7).

Homeless people also use high levels of health care, often in costly acute-care settings. One survey of homeless adults found that more than 40% used an emergency department (ED) at least once in the previous year (8); another study showed that although only 7.9% of patients using EDs were homeless, those homeless persons accounted for 54.5% of all ED visits (9). In this same survey of the homeless, 1 in 4 had been hospitalized in the previous year (1996) (10). The average length of an inpatient hospitalization for a homeless person was 36% longer than a nonhomeless person’s hospitalization for the same problem (11). Not surprisingly, the more unstable the sheltering arrangement (eg, unsheltered street homeless, emergency sheltered homeless), the more likely a homeless person was to use an ED for health care (12).

Developing a population-based approach and increasing health care systems’ capacity to address the health care needs of the homeless have challenges. Homeless people face multiple barriers to health care, including transportation, limited availability and fragmentation of health care services, difficulty scheduling and keeping appointments, perceived or actual stigma of homelessness, lack of trust, social isolation, and competing sustenance needs (13–15). Homeless people frequently have multiple acute health care needs, creating a triaging dilemma that can preclude addressing root-cause needs related to their homeless state (eg, exposure to violence, trauma, or the elements; untreated or undertreated physical or mental health conditions). Aligning and coordinating the resources needed to care for homeless people is difficult in traditional health care settings.

For homeless people and for other socially disadvantaged populations, effective health care often lies outside the confines of a strictly medical approach (1) and includes broader social determinants of health, such as housing, income, and family supports. Where and how health care delivery fits into this broad approach is unclear although a more expansive “medical home” construct provides a useful framework for considering health care delivery to homeless people and other vulnerable populations. The medical home is a proactive, primary care-based, interdisciplinary team model constructed on the principles of patient centeredness, a team-based, whole-person orientation to longitudinal care, and active communication and coordination among providers. This model is considered effective for patients with complex health care needs. This article describes the development of the Veterans Health Administration’s (VHA’s) national medical home model for homeless veterans, core principles and elements of the model, and key features of high-performing sites. We describe the development and implementation of the model over 4 years and present a pre-enrollment–post-enrollment analysis of health care use by homeless veterans participating in this model program.

## Methods

### Clinical model

The homeless medical home initiative —known at VHA as the Homeless Patient Aligned Care Team (H-PACT) program — was launched in 2011 as part of the Ending Homelessness Among Veterans initiative (16). The intent was to integrate and coordinate health and social services care for homeless veterans with a focus on the highest-risk, highest-need veterans unable or unwilling to access traditional health care. The program’s goals are to engage the patient in health care, stabilize them clinically, provide them with needed social services and programs, and expedite their placement in housing (Figure). The model draws heavily from lessons learned from the Health Care for the Homeless program funded by the US Department of Health and Human Services (17), the theoretic framework of the Behavioral Model for Vulnerable Populations (18), and homeless adaptations of both the chronic care model (19) and the ambulatory intensive care model (20). As homeless veterans stabilize clinically and socially, as evidenced by their moving into permanent housing and demonstrating appropriate self-care and health-seeking behaviors, they are transitioned to traditional care settings to continue their care.

**Figure:**
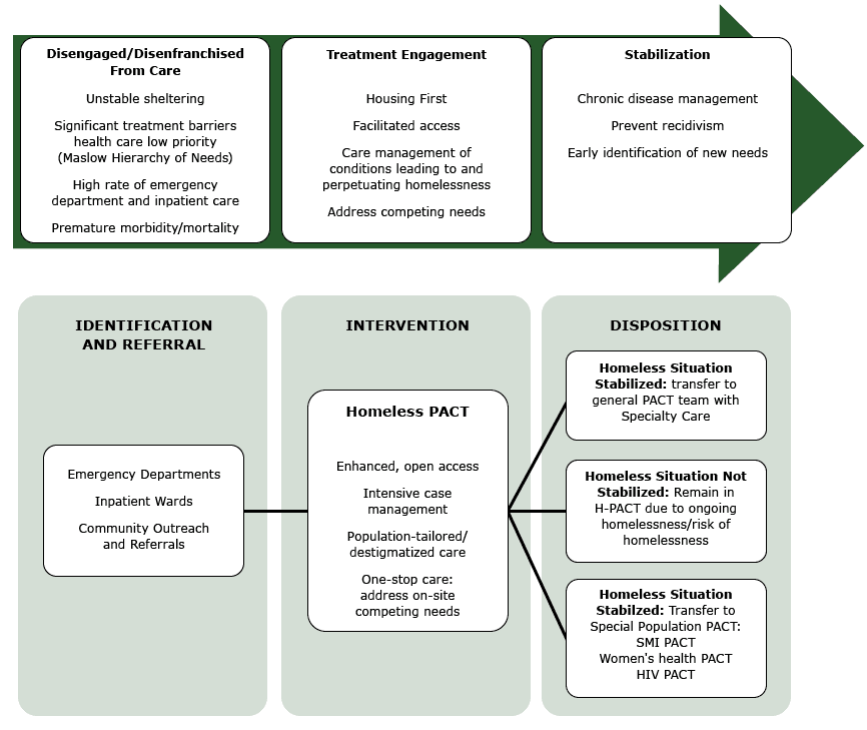
Homeless-patient aligned care team model for treatment engagement. Abbreviations: PACT, patient aligned care team, SMI, serious mental illness; HIV, human immunodeficiency virus.

Five core elements of the H-PACT model distinguish it from traditional primary care: 1) enhanced, low-threshold access to care with open-access, walk-in capacity, flexible scheduling (ie, patients do not need an appointment to be seen by their care team; H-PACTs have walk-in hours, and when a veteran has a scheduled appointment, it is usually for a time with latitude to accommodate late arrivals because of transportation or other issues), and clinical outreach to homeless people on streets, in shelters, and in community locations (eg, soup kitchens, community resource centers); 2) integrated services (ie, mental health services and primary care services are located close to each other, and providers from both services are involved in patients’ health care plan). In addition, sustenance needs (eg, food or food vouchers, hygiene kits, clothes, bus passes, other transportation assistance) are available at the same location*;* 3) intensive health care management that is integrated with community agencies with an emphasis on ongoing, continuous care; 4) ongoing staff training and development of homeless care skills; and 5) data-driven, accountable care processes. Implementation fidelity among sites is addressed in 2 ways. First, during start-up, teams are provided an itemized checklist specific to the H-PACT model (Table 1). Second, H-PACTs are asked to complete an annual survey querying how core elements are being carried out in their day-to-day practice. Surveys assess 1) access to care strategies, 2) team capacity and availability, 3) integration of homeless monitoring (a process for tracking veterans’ housing status and factors affecting it and incorporating information on status into clinical notes available to the entire health care team) and health care interventions into planning, 4) integration of social services and supports (ie, food assistance, clothing, hygiene, transportation) into on-site care, 5) and teams’ work in community outreach.

**Table 1:** Implementation Check List, Homeless Patient Aligned Care Team (H-PACT) Veterans Health Administration Clinics, October 2013–March 2014

Task	Implementation Objective
**Setting up the H-PACT team**	
Identify H-PACT team	Enabling team building, duty delegation, development of communication strategies, and care coordination across team members.
Skill development for team members	Providing comprehensive and integrated care to the homeless veteran that is directed to both clinical and housing objectives, which requires new skills and knowledge. Time should be allocated for the H-PACT team to be a learning organization with full participation expected for cyber seminars and other venues as appropriate.
**Incorporating the team within your local facility**	
Homeless veterans identification and referral process	Ensuring that homeless veterans most in need are referred to the H-PACT program and that the H-PACT is integrated into the rest of the medical facility and with community agencies interacting with homeless veterans.
Entry and exit criteria and process for H-PACT enrollment	Having clear criteria for who is assigned to the H-PACT team and when they can be transferred to a general population PACT. Criteria should be based on both homelessness and imminent risk of becoming homeless (including those recently housed) and on clinical needs that are not able to be addressed in a general population PACT. The team should have a process for meeting regularly to discuss these criteria and how they apply to patients enrolled in the program.
Integration, coordination with rest of medical center/facility care sites	Developing local service agreements and other arrangements that detail how care will be coordinated with the rest of the medical facility. Homeless veterans enrolled in the H-PACT program will have care needs that extend beyond that care team and may also face challenges navigating the system for that care.
**Developing patient flow and clinical care processes**	
Develop a patient orientation/activation program	Helping homeless veterans navigate the health care system, the H-PACT, and determining what care needs should be addressed can prevent inappropriate ED use, deferring care, and poor outcomes.
Access process: on-demand care and scheduled and planned care	Modeling programs to remove scheduling obstacles that prevent homeless veterans from keeping appointments. Programs should consider having 50% to 70% of all appointment slots available for walk-in and a small percentage set aside for scheduled care; walk-in and scheduled slots should be available for all team members, not only PCPs.
Clinic flow and patient flow	Developing processes so veterans do not get los” in the system as they navigate H-PACT care as well as referred care. Attention needs to be placed on wait times and competing demands (eg, bus schedules, soup kitchen and shelter check-in times).
Competing sustenance needs	Creating a one-stop model of care where multiple, often competing needs can be addressed to reduce the likelihood that a veteran will have to choose between getting care and pursuing other, more pressing needs.
Case management	Coordinating multiple care needs both to ensure that veterans get all concurrent needs addressed and that team members work efficiently and effectively together; having a designated process for triaging and discussing cases within the team.
Homeless-tailored care strategy	Conducting comprehensive assessments that incorporate intake assessment data and provide a vehicle for task assignments and tracking. Care needs are multifaceted and cross many disciplines.
Team meetings, huddles	Having a process whereby each H-PACT meets either before or after a clinic session to discuss patients seen (or to be seen) that day and to discuss care plans.
Community engagement	Partnering with community agencies that provide housing and social services or interact with homeless veterans outside of the clinic to allow the team 1) to better incorporate social determinants of health into the clinical model , 2), to have more effective 2-way communication, and 3) to better monitor the needs of homeless veterans outside of clinic visits.
Templated notes, note titles	Identifying and monitoring elements of care specific to homelessness in clinic notes. The use of template notes helps ensure consistent collection and monitoring of this information (eg, sheltering status, food security, safety, social networks).
Disease-specific management protocols (ie, addiction, mental health, chronic disease)	Developing program-wide, population-specific best practices and clinical protocols associated with improved clinical outcomes and more efficient health services use triggered by a specific diagnosis.
Use-specific management protocols or preventing ED use and for post-hospitalization care	Population-specific best practices/clinical protocols associated with improved clinical outcomes and more efficient health services use triggered by a specific health service utilization pattern will need to be developed for program-wide implementation
Emergency and crisis protocols (ie, potentially violent or suicidal patient, inclement weather)	Developing scenario-specific care strategies and plans to assist clinic staff, improve clinical outcomes, and create more efficient use of health services.
**Monitoring outcomes**	
Use of performance measures and clinical outcomes data	Identifying specific staff responsible for data retrieval and review, setting aside time in team meetings to discuss them, and assigning responsibility for specific outcomes. Developing a process of outcomes accountability is critical to H-PACT functioning.
Tracking care offsets	Demonstrating any value-added capacity created by the H-PACT model.
Tracking housing status	Demonstrating any value-added capacity created by the H-PACT model.

Abbreviations: PACT, patient aligned care team; PCMM, patient care management module; ED, emergency department; PCP, primary care provider.

Monthly site-specific reports are provided to each team and consist of the following monitored performance measures across key indicators: 1) panel growth (a measurement of a site’s ability to engage and retain homeless veterans in the care model); 2) patient complexity (measured by the Diagnostic Cost Group (DCG)-Index panel complexity score, a global composite score on a scale of 0-to-5 defined by key diagnoses (eg, diabetes, hypertension, congestive heart failure, depression, anxiety, post-traumatic stress disorder, addiction) and service use patterns (the higher the score, the more complex the veteran’s health problems) (21) to ensure that high-risk, highest-need veterans are being enrolled); 3) average number of visits per patient in each ambulatory care service or program (a measurement of treatment engagement in primary care and leveraging of H-PACT care to access specialist care); and 4) net reductions in ED use and hospital admissions (a comparison of a homeless person’s ED use in the 6 months before enrollment with that person’s use 6 months post-enrollment).

### Data collection and analysis

We collected demographic data on all homeless veterans who were enrolled in H-PACT as of August 1, 2014. This cut-off date was selected to correlate with the annual survey data submitted by each clinic. Health services use was identified through administrative records capturing clinical encounter data for each care visit for VA-specific types of health care use (primary care provider or other health care team members, specialty care visits, mental health, homeless programming [eg, case manager visits, placements in VHA-supported housing programs], acute care hospitalizations, and ED visits) and averaged over 12 months for people continuously enrolled during that time. Because of the high numbers of administrative records on clinical encounters associated with mental health visits and homeless program participation, we examined only the proportion of patients receiving those services post-enrollment. Also, non-VHA–based care was not considered in this analysis.

Analyses of pre-enrollment and post-enrollment use of health services were limited to VA-based inpatient hospitalizations and ED visits during the 6 months before enrollment in the H-PACT and the 6 months after enrollment. To provide historic context to our findings, we chose this period to be consistent with previous studies of homeless health services that used this timeframe. We included all veterans who enrolled in an H-PACT from October 2013 through March 2014, capturing data on their 6 month pre-enrollment use beginning in May 2013 (for October 2013 enrollees) and their 6 month post-enrollment use ending in August 2014 (for March 2014 enrollees). We stratified care teams into high performing, moderate performing, and low performing on the basis of the net change in their 6 month pre-enrollment–post-enrollment acute care use by patients enrolled in each site’s H-PACT program. High-performing H-PACTs were those demonstrating significant reductions in use of acute care services (>30% reduction in ED use or >20% reduction in hospitalizations). Moderate performing H-PACTs had a 0% to 30% reduction in ED use or a 0% to 20% reduction in hospitalizations. Low-performing H-PACTs had an increase in ED use or hospitalizations in the 6-months post-enrollment. These criteria were selected as a surrogate measure for the ability of the H-PACT to successfully transition care from acute care settings to ambulatory care settings, presumably driven by reducing the need for acute care, having the capacity to provide on-demand outpatient care, improvements in the patient’s physical environment, or the result of case management. The stratification was limited to well-established H-PACT teams at the 33 sites studied, which had been in operation for at least 18 months and had at least 100 patients enrolled to minimize potential biases associated with start-up or extremely small enrollments.

We used results from the 2014 clinic surveys to define characteristics and care elements of stratified sites: 1) access to care characteristics, 2) staffing and team member availability, 3) care management and treatment engagement approaches, 4) integration of homeless-specific interventions and monitors into clinical care, 5) incorporation of social support into care delivery, and 6) community integration elements.

### Statistical analyses

We conducted a 2-sample proportions analysis of low-performing and high-performing H-PACTs, comparing the proportion of stratified clinics with each care element described previously. Included in this analysis were subelements specific to a particular service or offering. We reported z test statistical values for all implementation variables and considered *P* ≤ .05 significant. We used STATA 8.0 (STATA Corp) for our analyses.

The H-PACT program was launched in 2011 with 32 sites and expanded to 58 medical facilities and approximately 18,000 patients by 2015. The demographic and ambulatory care use data used in this analysis were for the August 2014 enrollment of 14,088 patients with corresponding characteristics of 33 established H-PACTs. The pre-enrollment and post-enrollment acute care use data were the aggregated clinical data of 3,543 veterans enrolled in an H-PACT program from October 2013 through March, 2014.

## Results

The average age of participants was 53.4 years; 8.8% served in the military after September 11, 2001, 4.0% were women, and 11.0% were 65 years or older. Their DCG-Index complexity score was 0.96. These findings contrast with those for the overall population enrolled in primary care at VHA at the time: average age was 63 years and average DCG-Index complexity score was 0.66.

### Use of clinical or social services

Patients continuously enrolled in care for at least 1 year (n = 5,935) in 2014 averaged 3.4 clinic visits with their primary care provider (PCP) and an additional 5.9 separate visits with other members of the H-PACT team (eg, nurse case manager, social worker, health technician) during the previous 12 months. They also averaged 1.5 visits in a specialty clinic, and 82.2% were actively receiving mental health and substance abuse services. By comparison, patients in general VHA primary care clinics averaged 1.8 PCP visits per year. More than 96% of patients enrolled in an H-PACT were concurrently receiving homeless program services, including permanent supportive housing placement and case management (a jointly run US Department of Housing and Urban Development and VHA program that uses Section 8 housing coupled with VHA case management to place homeless veterans in permanent housing with ongoing care management), transitional housing support (grant and per diem housing), outreach (Health Care to Homeless Veterans outreach teams), justice programs (veterans courts, veterans justice outreach) and vocational assistance (Table 2).

**Table 2:** Demographic Characteristics and Health Services Used by Homeless Veterans (N = 3,543) at 33 Homeless Patient Aligned Care Team (H-PACT) Veterans Health Administration Clinics, October 2013 – March 2014

August 2014 Enrollment Data	
Total number enrolled	14,088
Veterans of military service after September 11, 2001, %	8.8
Women, %	4.1
Aged >65 y, %	11.0
DCG intensity score, mean	0.95
No. of H-PACT PCP visits per patient, mean	3.4
No. of specialty care visits per patient, mean	1.5
No. of H-PACT visits (excluding PCP visits) per patient, mean	5.9
Patients receiving mental health/substance abuse treatment services, %	82.0
Patients enrolled in homeless programs, %	96.0
Pre-enrollment/post-enrollment change in ED use for homeless veterans enrolled from October 2013 through March 2014 (N = 3,543): pre-6 months H-PACT enrollment = 3,022 ED visits; post 6 months H-PACT enrollment = 2,447 ED visits, %	−19.0
Pre-enrollment/post-enrollment change in hospitalizations for homeless veterans enrolled from October 2013 through March 2014: pre-6 months H-PACT enrollment = 812 hospitalizations; post-6 months H-PACT enrollment = 530 hospitalizations, %	−34.7

Abbreviations: DCG, diagnostic cost group; PCP, primary care provider; ED, emergency department

Overall, 82% of H-PACT sites provided hygiene support (showers, hygiene kits, and laundry assistance), 76% % provided direct assistance for transportation, 55% % had a clothes pantry on-site, and 42% % provided meals or other food assistance (eg, food pantry, assistance with food stamp applications, cooking classes for those recently housed). Additionally, 39% of H-PACTs had peer mentors available on-site to assist with care navigation; 30% had vocational programs on-site, 27% processed benefits and disability claims on site, and 21% provided on-site legal aid.

### Pre–post 6-month enrollment in the use of acute care service

From October 2013 through March 2014, 3,543 homeless veterans enrolled in the H^-^PACT program (416­–733 per month). In the 6 months before their enrollment, participating veterans had 3,022 ED visits and 812 hospitalizations. In the 6 months after enrollment, they had 2,447 ED visits (19.0% reduction) and 530 hospitalizations (34.7% reduction). Since the program’s inception (54 months previously), the overall reduction in both ED use and hospital admissions averaged 25%.

Overall 17 sites were found to be high performing, 9 sites were mid performing, and 7 sites were low performing. Although several core elements and features of the H-PACT model had been implemented to varying degrees throughout the sites studied, the association of these elements and features with high performance was limited (Table 3). High-performing sites were significantly more likely than mid-performing or low-performing sites to track housing status in the clinic notes (82.4% vs 42.9%; *P* = .05), have more than 50% fulltime equivalent staffing of a clinic nurse (88.2% vs 14.3%; *P* = .005) and primary care provider (82.4% vs 42.9%; *P* = .05), to have social services and supports embedded in the clinic (transportation, 94.1% vs 28.6%, *P* = .008; to offer food assistance on-site (64.7% vs 14.3%; *P* = .02); to have a clothes pantry (76.5% vs 28.6%; *P* = .03), and to participate in community events such as health screenings (82.4% vs 28.6%; *P* = .01) or community outreach (94.1% vs 57.1%; *P* = .03).

**Table 3:** Characteristics of 33 Homeless Patient Aligned Care Team (H-PACT) Veterans Health Administration Clinics

Site-Specific Survey Data^a^	High-Performing Site: >30% Reduction in ED Use Pre-Enrollment Versus Post-Enrollment or >20% Reduction in Hospitalizations (n = 17), % (n)	Mid-Performing Site: 0%–30% Reduction in ED Use Pre-Enrollment Versus Post-Enrollment or 0%–20% Reduction in Hospitalizations (n = 9), % (n)	Low-Performing Site: Increase in ED Use or Hospitalizations Post-enrollment (n = 7), % (n)	*P* Value^b^
**Access**				
Available >20 hours/week	76.5 (13)	55.6 (5)	42.9 (3)	.11
After-hours care/consult available	76.5 (13)	55.6 (5)	42.9 (3)	.11
<14 days to access mental health services	76.5 (13)	55.6 (5)	57.1 (4)	.34
Multiple ways to access care	94.1 (16)	66.7 (6)	71.4 (5)	.13
**Team characteristics**				
>50 Full-time primary care provider	82.4 (14)	44.4 (4)	42.9 (3)	.05
>50 Full-time nursing	88.2 (15)	66.7 (6)	14.3 (1)	.005
>50 Full-time social worker	70.6 (12)	66.7 (6)	28.6 (2)	.06
Integrated homeless program staff	88.2 (15)	77.8 (7)	57.1 (4)	.09
**Care management**				
≥3 primary care visits/patient/year	64.7 (11) (4.4 visits/patient)	77.8 (7) (5.3 visits/patient)	57.1 (4) (2.9 visits/patient)	.73
≥1.5 specialty care visits /patient/year	29.4 (5) (1.3 visits/patient)	44.4 (4) (1.6 visits/patient)	0 (0) (1.1 visits/patient)	.11
**Homeless-specific care**				
Clinical protocols				
Post-ED/ Hospitalization	58.8 (10)	66.7 (6)	42.9 (3)	.48
Disease-specific care	52.9 (9)	44.4 (4)	28.6 (2)	.28
**Housing integrated into care plan**				
Integrated clinical notes	94.1 (16)	88.9 (8)	71.4 (5)	.13
Housing status tracking	82.4 (14)	66.7 (6)	42.9 (3)	.05
**On-site social supports**				
Transportation	94.1 (16)	77.8 (7)	28.6 (2)	.008
Food	64.7 (11)	22.2 (2)	14.3 (1)	.02
Clothes	76.5 (13)	33.3 (3)	28.6 (2)	.03
**Community integration**				
Clinical outreach^c^	94.1 (16)	55.6 (5)	57.1 (4)	.03
Community partnership^d^	64.7 (11)	33.3 (3)	28.6 (2)	.11
Host community events^e^	82.4 (14)	77.8 (7)	28.6 (2)	.01

Abbreviation: ED, emergency department.

a Site-specific survey data were collected as part of the 2014 annual survey of H-PACT sites on care elements.

b We reported *z* test statistical values comparing the proportion of high-performing sites to low-performing sites that were implementing the targeted variable and considered *P* ≤ .05 significant.

c Clinical outreach includes mobile medical teams and H-PACT care team visits to local shelters, housing programs, and community agencies.

d Community partnerships are formal and informal relationships with community-based organizations evidenced by scheduled joint meetings, shared care management, as well as an established Memorandum of Understanding (MOU) with the agency.

e Community events include regional homeless veteran Stand Down events, health fairs, and other health-oriented events.

## Discussion

Incorporating social determinants of health into clinical care is one approach to effectively engaging and managing the needs of vulnerable and disenfranchised patients (22). Evaluation of the H-PACT program suggests that integration of social support services and social determinants into a clinical care model for homeless veterans can be effective in delivering comprehensive care. In our study, social support interventions were grouped into 3 categories: 1) programming that addressed sustenance needs such as food security, hygiene, and clothing assistance that compete with the patient’s ability to prioritize health care needs and access health services; 2) programming that facilitated physical, mental, and social recovery and stabilization, such as housing assistance, legal aid, and vocational training and assistance with disability claims; and 3) programming that facilitated getting treatment by reducing stigma, hassle, and inconveniences associated with obtaining health care. The last category can include outreach to community agencies; mobile medical teams; peer mentors; transportation; and open-access, care-on-demand capacity. Taken together, they address several health disparities and barriers to care that homeless veterans face (13–15,23). Although the merits of all of these care elements appear self-evident, we found that only increased nursing and primary care provider coverage, incorporating housing status into clinic notes, on-site social support (eg, transportation, food, clothing), and community events and outreach were significant. More study is needed both to validate these findings and better understand from a patient perspective their impact on care engagement and health outcomes.

The pre-enrollment and post-enrollment analyses showed substantial reductions in acute care use in the 6 months after enrollment in the H-PACT program. This type of analysis is subject to potential regression-to-the-mean biases, because drops in acute care use could be explained by recovery from an acute event and health stabilization independent of the program. However, previous research of enrollment in ambulatory care models often showed at least an initial increase in acute care use post-enrollment, presumably by removing barriers to access and addressing delayed or deferred health care needs (19,24–26). Studies currently underway using control-group comparison arms will be able to address the relative role of a regression-to-the-mean bias more definitively.

In our study patients enrolled in H-PACTs reduced their acute care use much faster than patients described in other reports. Other studies of homeless interventions found that reductions often occurred 6 to 12 months after people were enrolled in the program (19,24,25). We suggest that our findings are driven by 2 main factors. First, having the capacity to address subacute needs on demand in ambulatory settings is critical. Enhanced access to primary, specialty, and mental health care and to case management is consistent with the approach taken in the ambulatory-care– intensive-care unit model (20) and with previous research on newly enrolled homeless veterans (27). Second, integrating social services and supports and housing resources and assistance into the clinical model provides a holistic approach to patient care and addresses underlying causes for much of acute care use. This finding is also consistent with reductions in acute care use noted in housing-first models where housing placement is not made contingent on achieving sobriety or other threshold events. (28,29).

Finally, developing and maintaining a homeless medical home model such as H-PACT presents challenges and costs. The model requires that leaders commit resources and understand that activities perceived as inefficiencies for clinics with high patient-visit rates and relatively low enrollment caps are necessary for reducing use of acute care. Sustaining the capacity to offer comprehensive care for those most in need, such as homeless veterans, also requires active panel management of those patients enrolled in the program, including the ability to advance patients to lower-intensity care settings when they have stabilized in terms of clinical, housing, and social needs.

Our study has several limitations and strengths. The findings are from 4 years’ experience with a national demonstration project that consists of widely distributed care sites and settings. In contrast to single-site studies in more controlled settings, this study assumes a naturalistic, observational approach that may be more realistic when considering what is replicable and generalizable. Although using administrative data from VHA electronic medical records facilitates a comprehensive capture of demographic and health care use data, these data do not allow us to comment on care outside the VHA system. The parameters used for identifying high-performing H-PACTs were narrowly defined and do not address other equally important measures, such as housing stability, satisfaction with health care, and chronic disease management, which were considered elsewhere (17,23). We also did not control for patient-level factors beyond average DCG-Index intensity scores when stratifying clinics, and further research is needed to formally consider these designations. Additionally, as noted earlier, the use of pre-enrollment and post-enrollment data introduces a potential regression-to-the-mean bias. Finally, our implementation survey data are subject to several biases, including a social desirability bias, and further validation is needed to draw firm conclusions.

In summary, findings from this pilot project suggest that high levels of patient engagement in health care, evidenced by enhanced use of health care and social services, were associated with a population-tailored medical home approach for homeless veterans. Critical elements associated with high-performing clinics are the robust incorporation of social determinant programs into clinical care delivery, dedicated staff time, and community integration.

## References

[1] Institutes of Medicine Committee on Health Care for Homeless People. Homelessness, health, and human needs. Washington (DC): National Academies Press; 1988.

[2] Argintaru N , Chambers C , Gogosis E , Farrell S , Palepu A , Klodawsky F , A cross-sectional observational study of unmet health needs among homeless and vulnerably housed adults in three Canadian cities. BMC Public Health 2013;13(1):577. 10.1186/1471-2458-13-577 23764199 PMC3691921

[3] Baggett TP , O’Connell JJ , Singer DE , Rigotti NA . The unmet health care needs of homeless adults: a national study. Am J Public Health 2010;100(7):1326–33. 10.2105/AJPH.2009.180109 20466953 PMC2882397

[4] O’Toole TP , Conde-Martel A , Gibbon JL , Hanusa BH , Fine MJ . Health care of homeless veterans. J Gen Intern Med 2003;18(11):929–33. 10.1046/j.1525-1497.2003.21209.x 14687279 PMC1494947

[5] Hibbs JR , Benner L , Klugman L , Spencer R , Macchia I , Mellinger A , Mortality in a cohort of homeless adults in Philadelphia. N Engl J Med 1994;331(5):304–9. 10.1056/NEJM199408043310506 8022442

[6] Hwang SW , Orav EJ , O’Connell JJ , Lebow JM , Brennan TA . Causes of death in homeless adults in Boston. Ann Intern Med 1997;126(8):625–8. 10.7326/0003-4819-126-8-199704150-00007 9103130

[7] Baggett TP , Hwang SW , O’Connell JJ , Porneala BC , Stringfellow EJ , Orav EJ , Mortality among homeless adults in Boston: shifts in causes of death over a 15-year period. JAMA Intern Med 2013;173(3):189–95. 10.1001/jamainternmed.2013.1604 23318302 PMC3713619

[8] Kushel MB , Perry S , Bangsberg D , Clark R , Moss AR . Emergency department use among the homeless and marginally housed: results from a community-based study. Am J Public Health 2002;92(5):778–84. 10.2105/AJPH.92.5.778 11988447 PMC1447161

[9] Hastings SN , Smith VA , Weinberger M , Schmader KE , Olsen MK , Oddone EZ . Emergency department visits in Veterans Affairs medical facilities. Am J Manag Care 2011;17(6 Spec No.):e215–23. 21756015 PMC6519060

[10] Kushel MB , Vittinghoff E , Haas JS . Factors associated with the health care utilization of homeless persons. JAMA 2001;285(2):200–6. 10.1001/jama.285.2.200 11176814

[11] Salit SA , Kuhn EM , Hartz AJ , Vu JM , Mosso AL . Hospitalization costs associated with homelessness in New York City. N Engl J Med 1998;338(24):1734–40. 10.1056/NEJM199806113382406 9624194

[12] O’Toole TP , Gibbon JL , Hanusa BH , Fine MJ . Utilization of health care services among subgroups of urban homeless and housed poor. J Health Polit Policy Law 1999;24(1):91–114. 10342256 10.1215/03616878-24-1-91

[13] O’Toole TP , Johnson EE , Redihan S , Borgia M , Rose J . Needing primary care but not getting it: the role of trust, stigma and organizational obstacles reported by homeless veterans. J Health Care Poor Underserved 2015;26(3):1019–31. 10.1353/hpu.2015.0077 26320930

[14] Wen CK , Hudak PL , Hwang SW . Homeless people’s perceptions of welcomeness and unwelcomeness in healthcare encounters. J Gen Intern Med 2007;22(7):1011–7. 10.1007/s11606-007-0183-7 17415619 PMC2219712

[15] Gelberg L , Gallagher TC , Andersen RM , Koegel P . Competing priorities as a barrier to medical care among homeless adults in Los Angeles. Am J Public Health 1997;87(2):217–20. 10.2105/AJPH.87.2.217 9103100 PMC1380797

[16] US Department of Veterans Affairs, Office of Public and Intergovernmental Affairs. Secretary Shinseki details plan to end homelessness for veterans. http://www.va.gov/opa/pressrel/pressrelease.cfm?id=1807. Accessed November 19, 2009.

[17] McMurray-Avila M . Organizing health services for homeless people: a practical guide. Nashville (TN): National Health Care for the Homeless Council; 1997.

[18] Gelberg L , Andersen RM , Leake BD . The Behavioral Model for Vulnerable Populations: application to medical care use and outcomes for homeless people. Health Serv Res 2000;34(6):1273–302. 10654830 PMC1089079

[19] O’Toole TP , Buckel L , Bourgault C , Blumen J , Redihan SG , Jiang L , Applying the chronic care model to homeless veterans: effect of a population approach to primary care on utilization and clinical outcomes. Am J Public Health 2010;100(12):2493–9. 10.2105/AJPH.2009.179416 20966377 PMC2978184

[20] California Health Care Foundation. How ambulatory intensive caring units can reduce costs and improve outcomes. http://www.chcf.org/publications/2011/05/ambulatory-intensive-caring-units. Accessed: August 3, 2015.

[21] Pope GC , Kautter J , Ellis RP , Ash AS , Ayanian JZ , Lezzoni LI , Risk adjustment of Medicare capitation payments using the CMS–HCC model. Health Care Financ Rev 2004;25(4):119–41. 15493448 PMC4194896

[22] Bircher J , Kuruvilla S . Defining health by addressing individual, social, and environmental determinants: new opportunities for health care and public health. J Public Health Policy 2014;35(3):363–86. 10.1057/jphp.2014.19 24943659 PMC4119253

[23] Kertesz SG , Pollio DE , Jones RN , Steward J , Stringfellow EJ , Gordon AJ , Development of the Primary Care Quality-Homeless (PCQ-H) instrument: a practical survey of homeless patients’ experiences in primary care. Med Care 2014;52(8):734–42. 10.1097/MLR.0000000000000160 25023918 PMC4620084

[24] Blue-Howells J , McGuire J , Nakashima J . Co-location of health care services for homeless veterans: a case study of innovation in program implementation. Soc Work Health Care 2008;47(3):219–31. 10.1080/00981380801985341 19042482

[25] McGuire J , Gelberg L , Blue-Howells J , Rosenheck RA . Access to primary care for homeless veterans with serious mental illness or substance abuse: a follow-up evaluation of co-located primary care and homeless social services. Adm Policy Ment Health 2009;36(4):255–64. 10.1007/s10488-009-0210-6 19280333

[26] Weinberger M , Oddone EZ , Henderson WG . Does increased access to primary care reduce hospital readmissions? Veterans Affairs Cooperative Study Group on Primary Care and Hospital Readmission. N Engl J Med 1996;334(22):1441–7. 10.1056/NEJM199605303342206 8618584

[27] O’Toole TP , Bourgault C , Johnson EE , Redihan SG , Borgia M , Aiello R , New to care: demands on a health system when homeless veterans are enrolled in a medical home model. Am J Public Health 2013;103(S2, Suppl 2):S374–9. 10.2105/AJPH.2013.301632 24148042 PMC3969111

[28] Larimer ME , Malone DK , Garner MD , Atkins DC , Burlingham B , Lonczak HS , Health care and public service use and costs before and after provision of housing for chronically homeless persons with severe alcohol problems. JAMA 2009;301(13):1349–57. 10.1001/jama.2009.414 19336710

[29] Sadowski LS , Kee RA , VanderWeele TJ , Buchanan D . Effect of a housing and case management program on emergency department visits and hospitalizations among chronically ill homeless adults: a randomized trial. JAMA 2009;301(17):1771–8. 10.1001/jama.2009.561 19417194

